# Pneumococcal Disease and the Effectiveness of the PPV23 Vaccine in Adults: A Two-Stage Bayesian Meta-Analysis of Observational and RCT Reports

**DOI:** 10.1038/s41598-018-29280-2

**Published:** 2018-07-23

**Authors:** Hamid Latifi-Navid, Saeid Latifi-Navid, Behdad Mostafaiy, Sadegh Azimzadeh Jamalkandi, Ali Ahmadi

**Affiliations:** 1 Molecular Biology Research Center, Systems Biology and Poisonings Institute, Baqiyatallah University of Medical Sciences, Tehran, Iran; 2 Department of Biology, Faculty of Sciences, University of Mohaghegh Ardabili, Ardabil, Iran; 3 Biosciences and Biotechnology Research Center (BBRC), Faculty of Advanced Technologies, University of Mohaghegh Ardabili, Namin, Iran; 4 Department of Statistics, Faculty of Sciences, University of Mohaghegh Ardabili, Ardabil, Iran; 5 Chemical Injuries Research Center, Systems Biology and Poisonings Institute, Baqiyatallah University of Medical Sciences, Tehran, Iran

## Abstract

The efficacy of PPV-23 vaccine on outcomes of pneumococcal disease in adults still remains controversial due mainly to the lack of consistency between the results obtained from observational studies(OSs) and those obtained from randomized controlled trials(RCTs). As a consequence, the complexity in the structure of evidence available, in turn, generates a challenge for combining disparate pieces of evidence quantitatively. In this regard, we used a hierarchical Bayesian inference-based evidence synthesis of RCTs and observational data using a two-stage approach (in addition to a traditional random-effects meta-analysis) to examine the effectiveness of PPV-23 in adults. To this end, 21 studies were included involving 826109 adult participants. By a two-stage Bayesian meta-analysis, which was directly used for combining studies of different designs, the overall log OR (95% credible interval) for IPDs was −0.1048 (−0.3920,−0.0250), indicating a significant protective effect of the vaccination against IPDs. No significant effect of PPV-23 was found on all-cause pneumonia, pneumococcal pneumonia, and death from pneumonia, which confirmed the results obtained by a traditional method followed by stratified and sensitivity analyses. The estimated overall log OR (95% credible interval) was −0.0002 (−0.0241,0.0142), −0.0002 (−0.0110,0.0122), and −6.3912 × 10^−5^ (−0.0219,0.0131), respectively. The PPV-23 vaccine might be effective in preventing the most severe invasive forms of pneumococcal diseases, but not effective in preventing other clinical outcomes, in the adult population of 18 years and older.

## Introduction

Approximately, 1.6 million deaths annually occur due to pneumococcal diseases worldwide, with the highest incidence rate in children under 2 years and adults over 65 years of age^1,2^. Therefore, pneumococcal invasive (such as bacteremia/sepsis and meningitis) and non-invasive infections (such as sinusitis and otitis media) still remain major public health problems with a high morbidity and mortality. In the US, for example, the mortality rate of pneumococcal pneumonia is 13–23% in the elderly, compared to 5–7% in the general population^3^.

There are two available pneumococcal preventive vaccines: the older Polysaccharide Vaccines (PPVs) that contain a purified capsular polysaccharide and the newer Conjugate Vaccines (PCVs) that include a conjugation of a carrier protein to capsular polysaccharide. The PPVs, which have been developed gradually from 2- to 23-valent vaccines, have been available since the early 1980s^4^. They are widely recommended for all individuals aged 65 years and older and adults who are at high risk for invasive pneumococcal diseases (IPDs), especially those with a history of a chronic lung disease, heart failure, chronic renal failure, chronic liver disease, diabetes mellitus, and asplenia/sickle cell disease. Furthermore, the immunocompromised people including HIV/AIDS or blood borne dyscrasias patients with cochlear implants or chronic cerebrospinal fluid leak, residents of nursing homes, and patients in long-term care facilities are high-risk populations who are recommended to receive the polysaccharide vaccine. However, the protection level, either in the elderly or at high-risk populations, remains a controversial issue^5^.

The 23-valent pneumococcal polysaccharide vaccine (PPV-23) includes 23 capsular serotypes (1, 2, 3, 4, 5, 6B, 7 F, 8, 9 N, 9 V, 10 A, 11 A, 12 F, 14, 15B, 17 F, 18 C, 19 A, 19 F, 20, 22 F, 23 F, and 33 F) which account for 72%^6^ to 95%^7^ of IPDs, depending on the geographic location^8^. Typically, IPDs affect sterile body sites including cerebrospinal fluid, pleural fluid, and blood stream. This is while vaccination strategies are aimed to give appropriate protection. On the other hand, the adult pneumococcal vaccination policy is very complex and cannot be fixed; it needs to be adaptive and flexible^9,10^. Vaccination with PPV-23 has some limitations, including (1) inability to induce immunological memory at any age—it activates only the T cell-independent antibody responses; (2) being poorly immunogenic in infants; (3) no effect on pneumococcal carriage; (4) decline in the proportion of vaccine-covered serotypes since the introduction of PCVs in children; (5) the unproven efficacy of combination use of PPV-23 and PCV-13; and (6) being costly if all eligible individuals are vaccinated^10,11^.

Although some evidence support protective efficacy of PPVs against IPDs^8,12^, their effectiveness in preventing pneumonias, mortality, and other pneumococcal infections are still controversial^13^. Our knowledge about the efficacy of PPV-23 in reducing the risk of IPDs in immunocompetent older adults comes from a number of case-control and cohort studies^14–23^. One reason why the efficacy of the vaccine remains controversial may be the lack of consistency between the results obtained from the observational studies (OSs) and those obtained from controlled trials. Empirical studies have shown that insufficient quality of clinical trials including inadequate allocation concealment or failure to blind patients, caregivers or those assessing outcomes, can lead to biases that may threaten the validity of the results and exaggerate treatment effects^24^. If multiple evidence from different sources is combined into a single pooled effect estimate, it may improve the assessment of clinical effectiveness^25^. However, the complexity in the structure of evidence available, in turn, generates a challenge for combining disparate pieces of evidence quantitatively. The Cochrane Collaboration advices to consider randomized controlled trials (RCTs) and OSs separately and not to pool the different study types into a single pooled effect estimate^26^. However, HTA agencies, such as the National Institute for Health and Care Excellence (NICE) in United Kingdom, do not restrict evidence synthesis to RCTs and often demand the identification of all the relevant sources of evidence^27^. Unique statistical approaches to combine randomized and non-randomized studies in clinical research are increasingly being published in recent years^25^. Of these, Bayesian hierarchical models (BHMs) have been the most popular method for combining disparate sources of evidence^25^.

In the present study, first data from all types of study design were combined into a single random-effects meta-analysis, and then stratified the analysis to explore the source of heterogeneity between the studies. Second, a powerful strategy called fully Bayesian hierarchical modelling in two stages^28^ was used to incorporate data from various study designs into a single meta-analysis. It contrasts with common BHM where a single step is applied to estimate all parameters simultaneously. Therefore, a full Bayesian analysis was presented at the level of each study and then the results were summarized by the posteriors resulting from Markov Chain Monte Carlo Simulation, MCMC (a powerful method when the study-specific data structures are complex)^28^. The aim was to obtain a more precise estimation of the efficacy of PPV-23 in protecting against different pneumococcal outcomes, including all-cause pneumonia, pneumococcal pneumonia, death from pneumonia, and bacteremia, septicemia or invasive pneumococcal diseases (IPDs) in the adult population aged 18 years and older, and thus enabling clinicians to draw firmer conclusions.

## Results

### Selected studies

**Figure 1:**
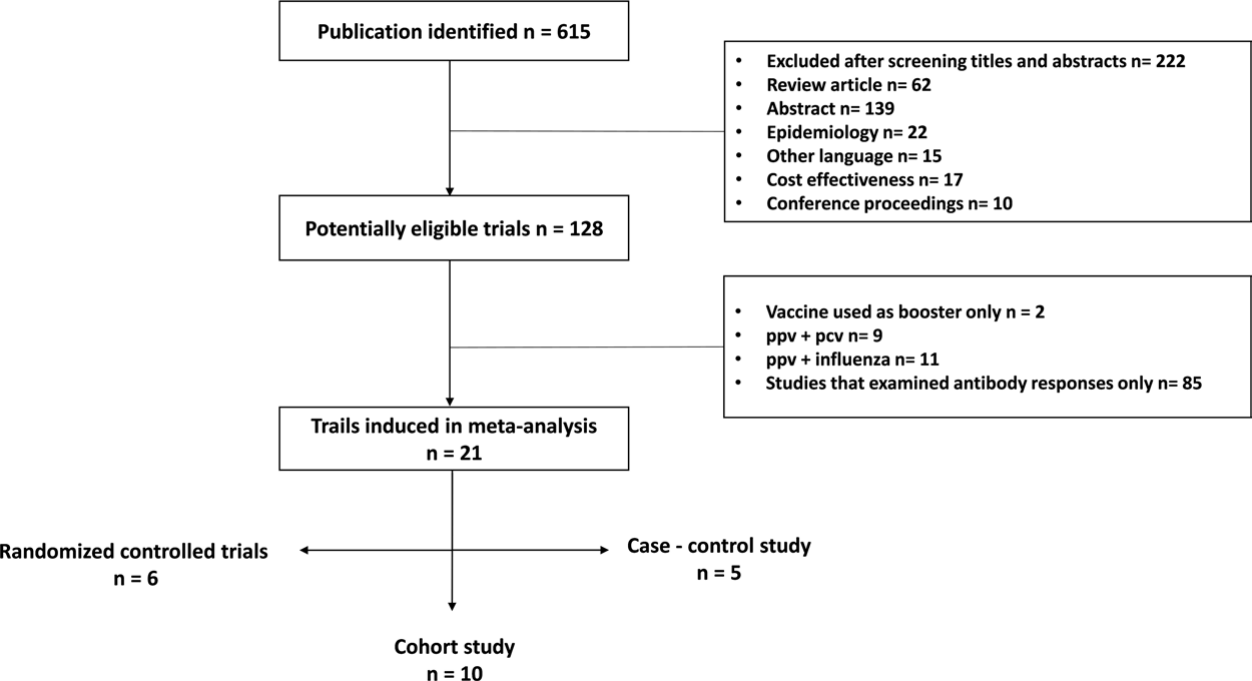
Identification and selection of eligible studies for inclusion in the meta-analysis.

The process of identifying eligible studies is clarified in Fig. 1. In the current study, 128 out of 615 references were considered to be potentially eligible. All the full texts of 128 publications were assessed. Among them, 107 were excluded from the final analysis; 2 described the use of vaccine as only a booster, 9 included simultaneous injection of PPV and PCV vaccines, 11 included simultaneous injection of PPV and influenza vaccines, and 85 only examined the antibody response. Finally, 21 studies were included in the meta-analysis involving 826109 adult participants, of which 6 (28.6%) were RCTs, 10 (47.6%) were cohort studies and 5 (23.8%) were case-control studies (Supplementary Tables S1, S2, and S3).

Among the six RCTs, five (83%) were described as double-blind, but placebo was used as a control. One (17%) was described as open and no intervention was used as a control. In this group, the follow-up time ranged from 1 to 6.7 years (mean 4.1 years); one trial (17%) did not provide information on the duration of follow-up. Two trials (33.3%) were performed in Uganda—a low income country—, one (16.7%) in Spain, one (16.7%) in Japan, one (16.7%) in Sweden and one (16.7%) in America. The study characteristics are shown in Supplementary Table S1.

In the cohort group, the follow-up time ranged from 3 to 10 years (mean 6 years); one study (10%) did not provide information on the duration of the follow-up. All studies in this group were performed in high income countries; six (60%) in Spain, two (20%) in Canada, one (10%) in Sweden, and one (10%) in China, Taiwan (Province of China). Supplementary Table S2 determines the features of cohort studies which were included in the meta- analyses.

In the case-control group, two studies (40%) were performed in the US, two (40%) in Spain, and one (20%) in Brazil. Supplementary Table S3 shows the characteristics of case-control studies that were included in the meta- analyses.

### Meta-analyses

Depending on the outcomes, 3 to 6 RCTs and 3 to 13 cohort/case-control studies were included in the meta-analyses. Each analysis included 2293 to 156812 precipitants for RCTs, and 173532 to 655106 precipitants for cohort/case-control studies.

### The effect of the PPV-23 vaccine on the relative risks of clinical outcomes according to the random-effects meta-analysis of combined study designs (RCTs and observational studies)

The pooled RR (95% CI) for all-cause pneumonia was 0.870 (0.692–1.092; *P* = 0.230). The quantity *I*^2^ of 74.335% (*P* = 0) was obtained, indicating that the degree of variability between studies was inconsistent with what would be expected to occur by chance alone. No evidence of publication bias was found; the *P*-value for Egger’s test was 0.972 (Table 1).

**Table 1:** Relative risks of all-cause pneumonia, pneumococcal pneumonia, death from pneumonia, and invasive pneumococcal diseases according to the combination of different study types (RCTs and observational studies) of the 23-valent pneumococcal polysaccharide vaccine in a random-effects meta-analysis.

Outcome	No. of studies	No. of participants (Vaccinated)	No. of cases	No. of participants (Unvaccinated or vaccinated with placebo)	No. of cases	Combined RR (95% CI)	Test for heterogeneity	Publication bias	
								Egger^’^s regression Intercept *P*	Begg’s *P*
All-cause pneumonia	9	88292	668	250071	3288	0.870 (0.692–1.092)	*I*^2^ = 74.335, *P* = 0.000	0.972	0.916
Pneumococcal pneumonia	10	8892	352	168729	530	0.952 (0.687–1.319)	*I*^2^ = 60.896, *P* = 0.006	0.869	0.858
Death from pneumonia	9	250252	761	257022	3186	0.538 (0.162–1.790)	*I*^2^ = 99.185, *P* = 0.000	0.564	0.602
Invasive pneumococcal diseases	17	247291	320	411308	1012	0.738 (0.595–0.916)	*I*^2^ = 70.182, *P* = 0.000	0.030	0.650

**Figure 2:**
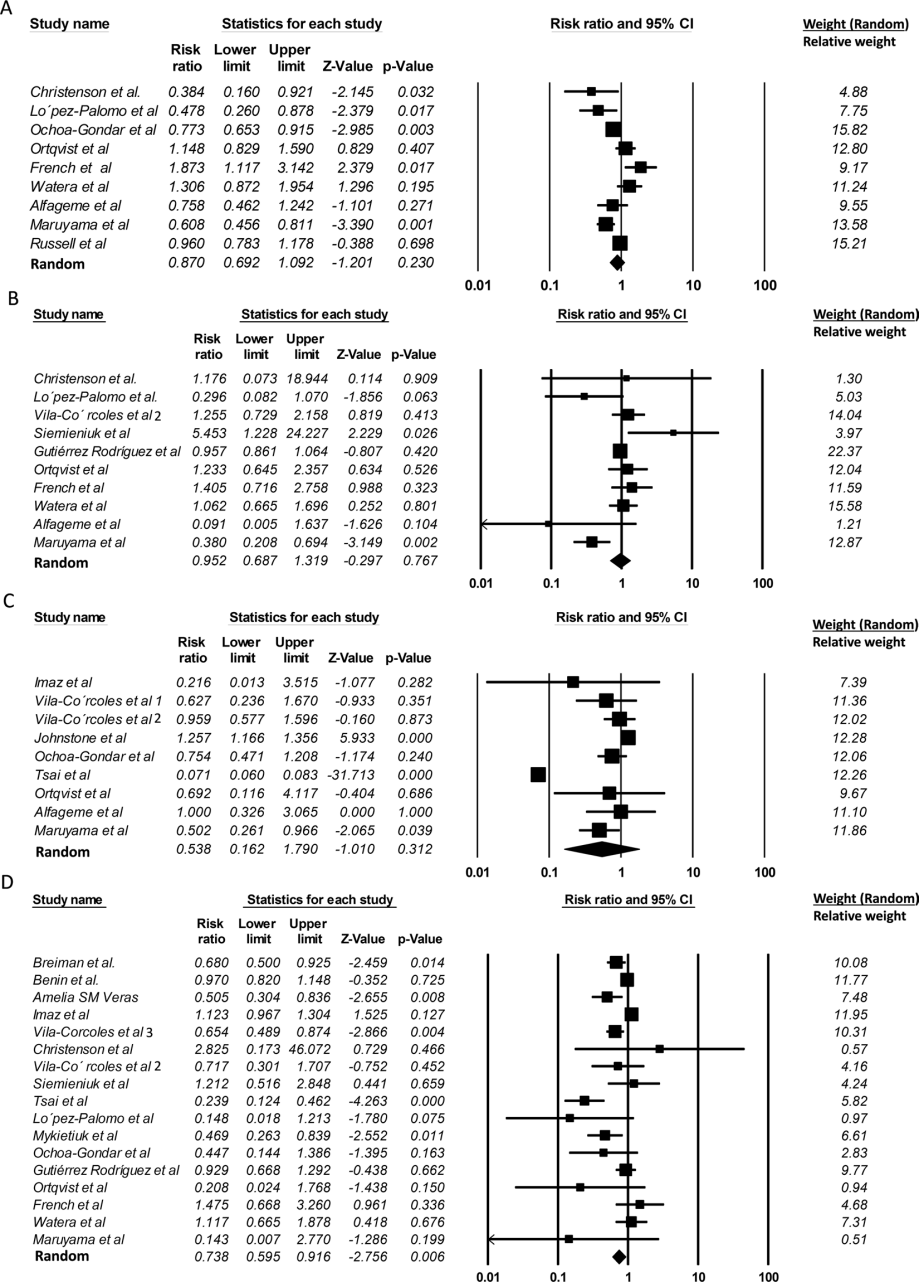
Summary plots of the random-effects meta-analyses of all studies (RCTs and observational studies) of the 23-valent pneumococcal polysaccharide vaccine for four clinical outcomes. The relative risk (squares, proportional to weights used in meta-analysis), with the summary measure and associated confidence intervals were determined for each defined group. (**A**) all-cause pneumonia, (**B)** pneumococcal pneumonia, (**C**) death from pneumonia, and (**D**); invasive pneumococcal diseases. Values less than 1 indicate a decreased risk of the outcomes, while values greater than 1 indicate an increased risk of the outcomes.

The pooled RR (95% CI) for pneumococcal pneumonia was 0.952 (0.687–1.319; *P* = 0.767). The Cochran’s Q test had a *P*-value of 0.006, which corresponded to the quantity *I*^2^ of 60.896%, indicating an evidence of heterogeneity between studies. No evidence of publication bias was found (Egger’s *P* = 0.869; Table 1). After the removal of studies conducted by Christenson *et al*., Lopez-Palomo *et al*., Siemieniuk *et al*., and Alfageme *et al*.^29–32^ with the largest variances (Fig. 2), the RR altered from 0.952 to 0.968 (95% CI; 0.727–1.287; *P* = 0.821), indicating the high stability of the results. However, there was still strong evidence of heterogeneity (Cochran’s Q test *P* = 0.035, *I*^2^ = 58.372%) but no publication bias was found (Egger’s *P* = 0.952).

The pooled RR (95% CI) for death from pneumonia was 0.538 (0.162–1.790; *P* = 0.312). The Cochran’s Q test had a *P*-value of 0, which corresponded to the quantity *I*^2^ of 99.185%, indicating strong evidence of heterogeneity between studies. No evidence of publication bias was found; the P-value for Egger’s test was 0.564 (Table 1). When the two studies of Imaz *et al*. and Ortqvist *et al*.^33,34^ with the largest variances (Fig. 2) were excluded, the results did not change substantially (RR = 0.567, 95% CI; 0.151–2.135, *P* = 0.402). However, there was still strong evidence of heterogeneity (Cochran’s Q test *P* = 0, *I*^2^ = 99.389%) but no publication bias was found (Egger’s *P* = 0.581).

The pooled RR (95% CI) in a random-effects model for bacteremia, septicemia or invasive pneumococcal diseases was 0.738 (0.595–0.916; *P* = 0.006). However, there was strong evidence of both the heterogeneity (Cochran’s Q test *P* = 0, *I*^2^ = 70.182%) and publication bias (Egger’s *P* = 0.030; Table 1), which might inflate summary relative risk estimates. When the four studies by Christenson *et al*., Lopez-Palomo *et al*., Ortqvist *et al*., and Maruyama *et al*.^30,31,34,35^ with the largest variances (Fig. 2) were excluded, the results did not change substantially (RR = 0.762, 95% CI; 0.615–0.944, *P* = 0.013). Significant heterogeneity (Cochran’s Q test *P* = 0, *I*^2^ = 74.426%) and publication bias (Egger’s *P* = 0.061) were still detectable across studies. The RRs varied between 0.697 (95% CI; 0.553–0.879, *P* = 0.002) and 0.804 (95% CI; 0.664–0.973, *P* = 0.025), by a leave-one-out sensitivity analysis (Table 2). The estimated effect size was statistically significant in all of the analyses in the presence of significant heterogeneity and publication bias. The *I*^2^ ranged from 60.446% to 72.018%. Only after the removal of Imaz *et al*.’s study, the test for bias was no longer statistically significant (Egger’s *P* = 0.106). Although there was still strong evidence of heterogeneity (Cochran’s Q test *P* = 0, *I*^2^ = 62.557%), the RR altered from 0.738 to 0.697 (95% CI; 553–879; *P* = 0.002; Table 2).

**Table 2:** Meta-analysis of the effectiveness of PPV-23 on invasive pneumococcal diseases, including all the types of study design: “Leave-One-Out” sensitivity analysis.

Studies	Random-effects model	*P*	Test for heterogeneity	*τ* ^2^	Publication bias *P*	
	RR (95% CI)		*I* ^2^		Egger^’^s regression Intercept *P*	Begg’s *P*
Breiman *et al*.	0.743 (0.589–0.937)	0.012	70.197	0.102	0.033	0.685
Benin *et al*.	0.701 (0.541–0.909)	0.007	71.395	0.139	0.043	1.000
Amelia S. M. Veras *et al*.	0.764 (0.613–0.951)	0.016	69.113	0.089	0.046	0.620
Imaz *et al*.	0.697 (0.553–0.879)	0.002	62.557	0.098	0.106	0.499
Vila-Corcoles *et al*. 1	0.747 (0.594–0941)	0.013	69.223	0.099	0.029	0.620
Christenson *et al*.	0.732 (0.589–0.910)	0.005	71.702	0.097	0.013	0.444
Vila-Corcoles *et al*. 2	0.738 (0.590–0.922)	0.008	71.913	0.099	0.035	0.752
Siemieniuk *et al*.	0.721 (0.577–0.901)	0.004	71.788	0.099	0.020	0.685
Tsai *et al*.	0.804 (0.664–0.973)	0.025	60.446	0.060	0.050	0.892
López-Palomo *et al*.	0.751 (0.607–0.930)	0.009	70.497	0.091	0.053	0.620
Mykietiuk *et al*.	0.765 (0.615–0.951)	0.016	69.269	0.089	0.049	0.685
Ochoa-Gondar *et al*.	0.749 (0.602–0.932)	0.010	71.267	0.095	0.045	0.752
Gutiérrez Rodríguez *et al*.	0.716 (0.564–0.907)	0.006	72.018	0.109	0.034	0.752
Ortqvist *et al*.	0.748 (0.603–0.928)	0.008	71.082	0.094	0.047	0.620
French *et al*.	0.713 (0.572–0.890)	0.003	71.216	0.096	0.015	0.558
Watera *et al*.	0.713 (0.567–0.895)	0.004	71.665	0.101	0.024	0.752
Maruyama *et al*.	0.745 (0.600–0.924)	0.007	71.258	0.095	0.045	0.685

### The effect of the PPV-23 vaccine on the relative risks of clinical outcomes according to the random-effects meta-analysis of RCTs

Among clinical trials, the pooled RR from a random-effects meta-analysis indicated no significant effect of PPV-23 on any of the clinical outcomes including all-cause pneumonia (RR = 1.009, 95% CI; 0.761–1.340; *P* = 0.948), pneumococcal pneumonia (RR = 0.837, 95% CI; 0.466–1.502; *P* = 0.550), death from pneumonia (RR = 0.606, 95% CI; 0.354–1.039; *P* = 0.068), and bacteremia, septicemia or invasive pneumococcal diseases (RR = 0.969, 95% CI; 0.496–1.891; *P* = 0.926; Table 3). There was strong evidence of heterogeneity for all-cause pneumonia (Cochran’s Q test *P* = 0.001, *I*^2^ = 75.451%) and pneumococcal pneumonia (Cochran’s Q test *P* = 0.010, *I*^2^ = 70.023%), while it was not statistically significant for death from pneumonia (Cochran’s Q test *P* = 0.575, *I*^2^ = 0%) and bacteremia, septicemia or invasive pneumococcal diseases (Cochran’s Q test *P* = 0.197, *I*^2^ = 35.797%). There was no evidence of publication bias for all the clinical outcomes; the *P*-values for the Egger’s and Begg’s tests were more than 0.1 (Table 3).

**Table 3:** Relative risks of all-cause pneumonia, pneumococcal pneumonia, death from pneumonia, and invasive pneumococcal diseases according to the random-effects meta-analysis of the RCTs.

Outcome	No. of trials	No. of study participants	No. of cases	Combined RR (95% CI)	Test for heterogeneity	Publication bias	
						Egger’s regression Intercept *P*	Begg’s *P*
All-cause pneumonia	6	156812	857	1.009 (0.761–1.340)	*I*^2^ = 75.451, *P* = 0.001	0.478	0.259
Pneumococcal pneumonia	5	4089	187	0.837 (0.466–1.502)	*I*^2^ = 70.023, *P* = 0.010	0.511	0.806
Death from pneumonia	3	2293	56	0.606 (0.354–1.039)	*I*^2^ = 0.000, *P* = 0.575	0.533	1.000
Invasive pneumococcal diseases	4	3493	86	0.969 (0.496–1.891)	*I*^2^ = 35.797, *P* = 0.197	0.148	0.734

In the analysis for pneumococcal pneumonia, after removing Alfageme *et al*.’s study^29^ which had the largest variance, the RR altered from 0.837 to 0.908 (95% CI; 0.680–1.214; *P* = 0.515), indicating the high stability of the results. However, there was still strong evidence of heterogeneity between studies (Cochran’s Q test *P* = 0.012, *I*^2^ = 72.559%). When the two studies by Ortqvist *et al*., and Maruyama *et al*.^34,35^ with the largest variances were excluded from the group of IPDs, the result was still not statistically significant (*P* = 0.381)

### The effect of the PPV-23 vaccine on the relative risks of clinical outcomes according to the random-effects meta-analysis of the cohort/case-control studies

Among the cohort/case-control studies that compared the 23-valent pneumococcal vaccinated with unvaccinated events, the pooled RR from a random-effects meta-analysis indicated a significant effect of PPV-23 on all-cause pneumonia (RR = 0.598, 95% CI; 0.386–0.927; *P* = 0.022), with no evidence of heterogeneity between studies (Cochran’s *Q* test *P* > 0.1, *I*^2^ = 54.397%). The *P*-value for the Begg’s test was 0.296, while Egger’s test had a *P*-value of 0.033, suggesting a possible bias (Table 4). After removing Ochoa-Gondar *et al*.’s study^36^ using a leave-one-out sensitivity analysis, the RR altered from 0.598 to 0.445 (95% CI; 0.270–0.733, *P* = 0.001).

**Table 4:** Relative risks of all-cause pneumonia, pneumococcal pneumonia, death from pneumonia, and invasive pneumococcal diseases according to the random-effects meta-analysis of the cohort/case-control studies.

Outcome		No. of studies		No. of participants (Vaccinated)		No. of cases		No. of participants (Unvaccinated)		No. of cases		Combined RR (95% CI)	Test for heterogeneity	Publication bias	
		Case-control	Cohort	Case-control	Cohort	Case-control	Cohort	Case-control	Cohort	Case-control	Cohort	Cohort and case-control		Egger^’^s regression Intercept *P*	Begg’ *P*
All-cause pneumonia		—	3	—	9981	—	226	—	171570	—	2853				
	Total	3		9981		226		171570		2853		0.598 (0.386–0.927)	*I*^2^ = 54.397, *P* = 0.112	0.033	0.296
Pneumococcal pneumonia		—	5	—	6826	—	264	—	166706	—	431				
	Total	5		6826		264		166706		431		1.074 (0.636–1.815)	*I*^*2*^ = 57.295, *P* = 0.053	0.690	0.806
Death from pneumonia		1	5	23	249090	0	740	139	255729	13	3138				
	Total	6		249113		740		255868		3151		0.474 (0.104–2.152)	*I*^*2*^ = 99.490, *P* = 0.000	0.589	0.452
Invasive pneumococcal diseases		5	8	474	245049	177	97	1214	408369	625	347				
	Total	13		245523		274		409583		972		0.702 (0.555–0.887)	*I*^*2*^ = 75.072, *P* = 0.000	0.028	0.669

PPV-23 showed no significant effect on pneumococcal pneumonia (RR = 1.074; 95% CI; 0.636–1.815; *P* = 0.789; Table 4). There was an evidence of heterogeneity (Cochran’s Q test *P* = 0.053, *I*^2^ = 57.295%) but no evidence of publication bias was found (Egger’s *P* = 0.690). Even after removing Christenson *et al*.’s study^30^ which had the largest variance, the estimated effect size failed to reach statistical significance (RR = 0.967, 95% CI; 0.872–1.073; *P* = 0.532; Cochran’s Q test *P* = 0.025, *I*^2^ = 67.907%).

No significant effect of PPV-23 was found on death from pneumonia (RR = 0.474, 95% CI; 0.104–2.152; *P* = 0.333; Table 4). There was strong evidence of heterogeneity (Cochran’s Q test *P* = 0, *I*^2^ = 99.490%) but no evidence of publication bias was found (Egger’s *P* = 0.589). Even after removing Imaz *et al*.’s study^33^ which had the largest variance, the estimated effect size failed to reach statistical significance (RR = 0.523, 95% CI; 0.105–2.614; *P* = 0.430; Cochran’s Q test *P* = 0, *I*^2^ = 99.592%).

The pooled RR also showed a significant effect of PPV-23 on bacteremia, septicemia or invasive pneumococcal diseases (RR = 0.702, 95% CI; 0.555–0.887; *P* = 0.003), although evidence of pronounced heterogeneity was found between studies (Cochran’s *Q* test *P* = 0, *I*^2^ = 75.072%; Table 4). The P-value for the Begg’s test was 0.669, while the Egger’s test had a P-value of 0.028, suggesting a possible bias. When two studies by Christenson *et al*.^30^ and López-Palomo *et al*.^31^ with the largest variances (wide intervals) were excluded, the results did not change substantially (RR = 0.709, 95% CI; 0.562–0.896, P = 0.004), indicating the stability of the results. However, there was still a strong evidence of heterogeneity between studies (Cochran’s *Q* test *P* = 0, *I*^2^ = 77.624%). Moreover, the *P*-value for the Begg’s test was 0.275, while it was 0.015 for the Egger’s test, suggesting a possible bias. The RRs varied between 0.652 (95% CI; 0.507–0.838, *P* = 0.001) and 0.774 (95% CI; 0.631–0.951, *P* = 0.015), by a leave-one-out sensitivity analysis (Supplementary Table S4). Then, we conducted a stratified analysis by the study design. In group A, which included 5 case-control studies, the odds ratio (OR) of 0.620 (95% CI; 0.413–0.932; *P* = 0.021) was obtained. There was still evidence of heterogeneity among studies (Cochran’s *Q* test *P* = 0.044, *I*^2^ = 59.164%), but no evidence of publication bias (Begg’s *P* = 0.806; Egger’s *P* = 0.624) was found. In group B, which included 8 cohort studies, the RR was 0.581 (95% CI; 0.356–0.948; *P* = 0.030). There was evidence of heterogeneity among studies (Cochran’s *Q* test *P* = 0.006, *I*^2^ = 64.986%). The test for publication bias was no longer statistically significant (Begg’s *P* = 0.710; Egger’s *P* = 0.488).

### Estimation of overall log odds ratios of the clinical outcomes by combining different study types in a two-stage Bayesian hierarchical meta-analysis

The overall results were obtained as the median and 2.5th and 97.5th percentiles of the posterior distribution of the parameter mu. We did a random-effects meta-analysis where mu was the mean of the distribution of study treatment effects (the tau_i), which was assumed normally distributed. Estimates were expressed as posterior medians and 95% credible intervals for logarithm of odds ratio. No significant protective effect of the PPV-23 vaccine was found on all-cause pneumonia, where the estimated overall log OR (95% credible interval) was −0.0002 (−0.0241, 0.0142). The vaccination had a negative effect in the Ortqvist *et al*.’s study. In the other studies, the vaccination did not have any effect on the outcome (Table 5).

**Table 5:** Estimation of overall log odds ratios on all cause pneumonia, pneumococcal pneumonia, death from pneumonia, and invasive pneumococcal disease by combining different study types into a two-stage Bayesian hierarchical meta-analysis.

	Log Odds Ratio^a^	Median	Credible interval
All-cause pneumonia	Christenson *et al*.	−0.1493	(−0.4109, 0.1630)
	López-Palomo *et al*.	−0.0702	(−0.5136, 0.2675)
	Ochoa-Gondar *et al*.	−0.0174	(−0.3320, 0.2945)
	Ortqvist *et al*.	0.3797	(0.2199, 0.5430)
	French *et al*.	−0.1073	(−0.4558, 0.1866)
	Watera *et al*.	0.0100	(−0.1689, 0.6189)
	Alfageme *et al*.	−0.0150	(−0.4908, 0.4290)
	Maruyama *et al*.	−0.0088	(−0.4499, 0.4049)
	Russell *et al*.	−0.1647	(−0.6803, 0.0895)
	**Overall**	**−0.0002**	**(−0.0241, 0.0142)**
Pneumococcal pneumonia	Christenson *et al*.	−0.0270	(−0.0311, 0.3098)
	López-Palomo *et al*.	−0.1546	(−0.9943, 0.3090)
	Vila-Co´ rcoles *et al*.	0.0474	(−0.2121, 0.3743)
	Siemieniuk *et al*.	0.0589	(−0.2027, 0.5835)
	Gutiérrez Rodríguez *et al*.	−0.0582	(−0.2749, 0.1446)
	Ortqvist *et al*.	0.0386	(−0.2497, 0.3828)
	French *et al*.	0.0518	(−0.2269, 0.4205)
	Watera *et al*.	0.0168	(−0.2557, 0.2987)
	Alfageme *et al*.	−0.5410	(−0.7522, 0.0820)
	Maruyama *et al*.	−0.2069	(−0.6663, −0.0389)
	**Overall**	**−0.0002**	**(−0.0110, 0.0122)**
Death from pneumonia	Imaz *et al*.	0.0508	(−0.1493, 0.0688)
	Vila-Córcoles *et al*.	−0.0551	(−0.5107, 0.2731)
	Vila-Córcoles *et al*.	−0.0184	(−0.3339, 0.2845)
	Johnstone *et al*.	0.3675	(0.2035, 0.5336)
	Ochoa-Gondar *et al*.	−0.0974	(−0.4696, 0.1685)
	Tsai *et al*.	−0.0040	(−0.4310, 0.2840)
	Ortqvist *et al*.	−0.0201	(−0.5053, 0.3890)
	Alfageme *et al*.	−0.0019	(−0.4104, 0.3753)
	Maruyama *et al*.	−0.1844	(−0.6610, 0.1470)
	**Overall**	**−6.3912 × 10** ^**−5**^	**(−0.0219, 0.0131)**
Invasive pneumococcal disease	Breiman *et al*.	−0.4924	(−0.8869, −0.119)
	Benin *et al*.	−0.0901	(−0.4826, 0.3321)
	Amelia S. M. Veras *et al*.	−0.7102	(−1.2930, −0.1896)
	Imaz *et al*.	0.276	(−0.5237, 1.2620)
	Vila-Corcoles *et al*.	−0.6901	(−1.1930, −0.2248)
	Christenson *et al*.	−0.0667	(−1.1440, 0.7419)
	Vila-Córcoles *et al*.	−0.2410	(−0.9493, 0.4065)
	Siemieniuk *et al*.	0.0668	(−0.5979, 0.7863)
	Tsai *et al*.	−1.1040	(−1.7110, −0.5718)
	López-Palomo *et al*.	−0.6131	(−1.7180, 0.1738)
	Mykietiuk *et al*.	−0.7491	(−1.4230, −0.1722)
	Ochoa-Gondar *et al*.	−0.4900	(−1.3150, 0.2653)
	Gutiérrez Rodríguez *et al*.	−0.0851	(−0.4539, 0.2737)
	Ortqvist *et al*.	−0.5335	(−1.5480, 0.2790)
	French *et al*.	0.1938	(−0.4358, 0.8884)
	Watera *et al*.	0.0763	(−0.4174, 0.5816)
	Maruyama *et al*.	−0.6528	(−1.782, −0.5930)
	**Overall**	**−0.1048**	**(−0.3920, −0.0250)**

^a^Estimates were expressed as posterior medians and 95% credible intervals for logarithm of odds ratio.

The overall log OR (95% credible interval) for pneumococcal pneumonia was −0.0002 (−0.0110, 0.0122), indicating no significant effect of the vaccination on the outcome. The vaccination had a significant effect in Maruyama *et al*.’s study. In the other studies, the vaccination did not have any effect on the outcome (Table 5).

No significant protective effect of the PPV-23 vaccine was found for death from pneumonia, where the estimated overall log OR (95% credible interval) was −6.3912 × 10^−5^ (−0.0219, 0.0131). The vaccination had a negative effect in the Johnstone *et al*.’s study. In the other studies, the vaccination did not have any effect on the outcome (Table 5).

The overall log OR (95% credible interval) on invasive pneumococcal diseases was −0.1048 (−0.3920, −0.0250), indicating a significant protective effect of the vaccination against IPDs. This effect was seen in studies by Breiman *et al*., Amelia S. M. Veras *et al*., Vila-Corcoles *et al*., Tsai *et al*., Mykietiuk *et al*., and Maruyama *et al*. In the other studies, the vaccination did not have any effect on the outcome (Table 5).

## Discussion

There is a serious debate concerning the efficacy of the PPV-23 vaccine on clinical outcomes in adults. In this regard, a detailed meta-analysis was conducted including 826109 adult participants distributed into 21 studies with different types of study designs. Each study was involved according to the predefined eligibility criteria. In order to avoid manipulation of these criteria, we did not straightforwardly exclude outliers based on a statistical test of homogeneity. The analyses of either the combined study designs (RCTs and OSs) or observational studies showed that PPV-23 significantly reduced the incidence of IPDs, but not pneumococcal pneumonia and death from pneumonia. The RR ranged from 0.581 to 0.738. The only meta-analysis of cohort studies showed a significant protective effect of PPV-23 against all-cause pneumonia (RR = 0.598). No significant effect of PPV-23 on any of the clinical outcomes was found in the RCTs. When the analysis was restricted to high income countries, for all-cause pneumonia and pneumococcal pneumonia, although the RRs altered, the difference failed to reach significance (RR = 0.852, 95% CI; 0.644–1.128 for ACP and RR = 0.540, 95% CI; 0.178–1.641 for PP). What was remarkable in most analyses, whether all studies were combined or stratified by study designs, was the high levels of heterogeneity, ranged from 57.295% to 99.490%, that might undermine the credibility of the results. Although *I*^2^ does not depend on the number of studies or the effect measure used^37^, it depends on the size of the individual studies in the meta-analysis^38^. Given the large sample size of the individual studies (i.e., the within-study variation is small) *I*^2^ could then be large (like >75%), even if the between-study variation is small. This was therefore a problem particularly when the data of cohort studies were being analysed (having typically large sample sizes). Though any amount of heterogeneity was acceptable as the predefined eligibility criteria were sound and met^39,40^, we used a random-effects model, assuming a particular form to heterogeneity (often, but not necessarily, a normal distribution), to overcome it. In addition, controlling the outliers on the forest plot and performing sensitivity analyses^41^ which indicated the stability of the pooled RR estimates and a close correlation between *I*^2^ and *τ*^2^ (among-study variance)—that means excluding studies to reduce *I*^2^ resulted in reducing *τ*^2 ^^39,40^—reaffirmed the accuracy and power of the results. The next problem was dealing with the positive publication bias, which is often tricky^42,43^. The analysis of the PPV-23 vaccine effectiveness on all-cause pneumonia among cohort studies revealed potential sources of bias (*P* < 0.1 for Egger’s test). It can be misleading if we ignore between-study heterogeneity when assessing publication bias^43^. As the meta-analysis was not large (i.e., the number of studies was small (<10) for all-cause pneumonia), methods to test or adjust for publication bias in the presence of heterogeneity might not be powerful. In contrast, it might be a serious threat to the validity of meta**-**analysis for IPDs **(***P* = 0.028 for Egger’s test**)** as the analysis was done on 13 eligible cohort/case-control studies. However, after stratification by the study design, the potential bias was no longer statistically significant (Egger’s *P* = 0.624 and 0.488 for case-control and cohort studies, respectively) and was effectively ruled out, further strengthening the validity and robustness of the results.

Supplementary Tablesncies between the meta-analyses of RCTs and observational studies, largely influenced by the study design. The meta-analyses of RCTs failed to identify a significant overall protective effect of PPV-23 on clinical outcomes, whereas the others (observational studies) indicated a protective effect of PPV-23 against all-cause pneumonia and IPDs. Both RCT and observational study estimations of the PPV-23 vaccine effectiveness are significantly affected by the quality of the study designs and both classes of the study have strengths and weaknesses^44^. Residual confounding, which is a fundamental criticism of observational studies, is always a potential source of discrepancy between RCT and observational studies. Although randomization removes the chances of confounding, there are other biases inherent to RCTs, and the external validity of RCTs may be also limited^45^. In the present meta-analysis, however, the 5 out of 6 RCTs were described as double–blinding. This might be the most acceptable method to minimize biases caused by the placebo effect. However, it could not always provide us with the unbiased estimations of the effect. High quality scores (3–5 points on the Jadad scale) were assigned for all the included RCTs (Supplementary Table S5). Nine cohort studies had high quality scores (7 or 8 points) on the 9-star NOS, and one had a low score (6 points) (Supplementary Table S6). High quality scores (8 or 9 points on the 9-star NOS) were assigned for all the included case-control studies (Supplementary Table S7).

Most of the previous studies did not evaluate PPV-23 independently; they considered all valencies and were inconsistent due to several methodological flaws^8,46,47^. We not only evaluated PPV-23 independently, but also excluded studies which included simultaneous injection of PPV and PCV or PPV and IVs, and those in which the other valencies of the PPV vaccine or any other specific vaccine (i.e., PCV or IVs) were used as a control. Otherwise, it could influence the net impact of the 23-valent PPV vaccine on different outcomes and the assumption of the synergistic and additive effects of two vaccines on the outcomes could not be ruled out. However, Diao *et al*.’s study^48^ included different interventions in a final meta-analysis of RCTs, which caused the real effectiveness of the 23-valent PPV vaccine to be controversial.

The potential adverse effects of serotype replacement are still a big challenge when implementing routine childhood PCV programs. In particular, where the lost vaccine serotype carriage is almost consistently replaced by non-vaccine serotype carriage, the net effectiveness of a vaccine is less than expected, in general. Moreover, the degree of replacement in disease, which is resulted from replacement in carriage, depends on the invasive potential of the serotypes involved^49,50^. In the present study, based on the pre-defined exclusion criteria, a limited number of included studies were conducted following childhood PCV vaccination programs. When these studies were excluded, the results did not change substantially (data are not shown), which indicated the stability of the results. In contrast, most of the studies included in Kraicer-Melamed *et al*.’s meta-analysis of PPV-23 vaccine effectiveness on CAP and IPDs were conducted following PCV-7- or PCV-13-based childhood programs that have decreased the prevalence of PCV-7- or PCV-13-type pneumococcal serotypes in adults, respectively^51^.

By the use of two-stage Bayesian hierarchical meta-analysis, which was directly used for combining studies of different designs, the overall log OR (95% credible interval) for IPDs was −0.1048 (−0.3920, −0.0250), indicating a significant protective effect of the vaccination against IPDs. No significant effect of PPV-23 was found on all-cause pneumonia, pneumococcal pneumonia, and death from pneumonia, which confirmed the results obtained by a traditional method. The estimated overall log OR values (95% credible interval) were −0.0002 (−0.0241, 0.0142), −0.0002 (−0.0110, 0.0122), and −6.3912 × 10^−5^ (−0.0219, 0.0131), respectively. As mentioned by Lunn *et al*.^28^, two limitations exist in the two-stage Bayesian hierarchical modelling employed in this article. One is the assumption of marginal independence between studies in stage 1 and another is dealing with sparse data. It is worthwhile to note that both of these limitations did not occur here since our Metropolis sampler was non-degenerate in stage 1 and the data was not sparse.

In conclusion, the PPV-23 vaccine might be effective in preventing the most severe invasive forms of pneumococcal diseases, but not effective in preventing other clinical outcomes, in the adult population of 18 years and older. It was validated by a fully Bayesian hierarchical modelling in two stages, which provides a high-performance approach to more complex evidence syntheses, such as multi-parameter evidence synthesis or mixed treatment comparisons. Although the meta-analysis of cohort studies, but not RCTs, indicated a significant protective effect against all-cause pneumonia, the number was not large and thus the results should be interpreted with caution. The high-quality large RCTs are required to more confidently validate the efficacy of the PPV-23 vaccine in protecting against all-cause pneumonia.

Determining the most common circulating pneumococcal serotypes in the community, designing more RCT and analytical studies to evaluate the effectiveness of pneumococcal vaccines, and finally evaluating the effectiveness of PCVs administration along with PPV23 can help to effectively measure the protective effect of PPV23. Regarding the cost benefits of vaccination against IPD compared to the antibiotic therapy strategies, and due to the increasing rate of IPD worldwide, the vaccination of the elderly is recommended.

## Methods

### Data sources

We searched MEDLINE, EMBASE, LILACS (Latin American and Caribbean Health Sciences Literature), AIM (African Index Medicus), and IndMed (Indian Medlars Centre) databases by using the keywords “23-valent pneumococcal vaccine”, “PPSV-23”, or “PPV-23” in combination with “outcomes”, “clinical outcomes”, or “clinical effectiveness”. These keywords were combined with terms describing the study design; “randomized-controlled trial”, “controlled clinical trial”, “clinical trial”, “cohort study”, and “case-control study”. We attempted to include all randomized comparisons of PPV-23 with a placebo in any type of population.

### Inclusion and exclusion criteria

We included RCTs that compared the PPV-23 vaccine with a placebo or no intervention, and cohort studies and case-control studies that compared outcomes between pneumococcal polysaccharide vaccinated and unvaccinated groups. Only English language articles were applied in this study. We excluded uncontrolled and observational intervention studies; animal and laboratory studies; studies examining antibody responses only; studies in which the PPV-23 vaccine was used as a booster after vaccination with conjugate pneumococcal vaccine; studies in which the other valencies of the PPV vaccine or any other specific vaccine (i.e., PCV or influenza vaccines & IVs) were used as a control; studies that included simultaneous injection of PPV and PCV vaccines, and those including the simultaneous injection of PPV and IVs; studies performed in child populations; the results coming from abstracts only; and review articles. Data from each potentially relevant article were extracted independently by two investigators (H.L.-N. and S.L.-N.) and discussed to solve any disagreement. Moreover, the information on the methodologic quality of the studies was extracted. The observed interrater agreement was measured using the kappa statistic. Finally, 21 original papers were left for analyses, which were divided into three groups according to their study design: RCTs^29,34,35,52–54^, cohort studies^20,30–32,36,55–59^, and case-control studies^14,15,33,60,61^ (Supplementary Tables S1, S2 and S3; Fig. 1). More detailed information about how pneumonia is diagnosed (for example by clinical features such as cough, fever, and pleuritic chest pain, or by chest X-ray or another method) and how pneumococcal pneumonia is confirmed (for example by cultivation or PCR-based methods on different clinical samples such as bronchoalveolar lavage, naso-pharyngeal**-**throat swabs and nasal swabs**)** is summarized in Supplementary Table S8. Quality assessment of RCTs and observational studies was determined on the basis of Jadad^62^ and Newcastle-Ottawa (NOS)^63^ quality assessment scales, respectively.

### Meta-analysis Outcomes

The following four outcomes were considered: (a) all-cause pneumonia; (b) pneumococcal pneumonia; (c) death from pneumonia; and (d) bacteremia, septicemia or invasive pneumococcal diseases (defined as isolation of *S. pneumoniae* from sterile body fluids).

### Classical meta-analysis method

Combined relative risk (RR) and 95% confidence intervals (CIs) were estimated for each study. The significance of pooled RR was tested by *Z*-test, and a *P*-value less than 0.05 was considered as statistically significant. The Cochran’s *Q* statistic was used to test heterogeneity among the included studies. The DerSimonian and Laird random-effects^64^ model was used to conduct the meta-analyses. Besides, we examined the *I*^2^ (I-squared) statistic describing the amount of variations due to true differences (heterogeneity) rather than random errors. The *I*^2^ values of 25%, 50%, and 75% corresponded to the low, moderate, and high levels of heterogeneity^40^, respectively. To explore the source of heterogeneity, we performed subgroup analyses according to the types of study design. Furthermore, the studies which had both the largest variance (wide intervals) and the extreme outlier weight in each clinical outcome group were identified. Then, a leave-one-out sensitivity analysis was conducted to assess the impact of individual studies, and thus the average RR was estimated in the absence of each study and heterogeneity was quantified using both the *I*^2^ and *τ*^2^ statistics^40^. The Egger’s regression asymmetry test and the Begg-Mazumdar adjusted rank correlation test were used to statistically assess the potential publication bias^65,66^. *P*-values less than 0.10 indicated the presence of bias. All the analyses were performed using a Comprehensive Meta-Analysis software (version 2, Biostat, Englewood, NJ), followed by PRISMA guidelines^67^, and 2-tailed *P*-values were calculated in all the tests.

### Specifications for a two-stage Bayesian hierarchical model

Let ${y}_{{C}_{i}}$ and ${y}_{{T}_{i}}$ denote the number of control and treatment groups of study $i,$ respectively. In addition, let ${\pi }_{{C}_{i}}$ and ${\pi }_{{T}_{i}}$ represent the corresponding underlying probabilities (the probability of observing the disease again) of these groups. The total number of individuals in these groups are ${n}_{{C}_{i}}$ and ${n}_{{T}_{i}}$. We have

1 $${{y}}_{{{C}}_{{i}}} \sim \,{Binomial}({{n}}_{{{C}}_{{i}}},{{\pi }}_{{{C}}_{{i}}}),{{y}}_{{{T}}_{{i}}} \sim {Binomial}({{n}}_{{{C}}_{{i}}},{{\pi }}_{{{C}}_{{i}}}){i}=1,\ldots ,{N}.$$

Consider the model

2 $${logit}({{\pi }}_{{{C}}_{{i}}})={{\xi }}_{{i}}-\frac{{{\theta }}_{{i}}}{2},\,{logit}({{\pi }}_{{{T}}_{{i}}})={{\xi }}_{{i}}+\frac{{{\theta }}_{{i}}}{2},\,{i}=1,\ldots ,{N},$$

where ${\xi }_{i}s$ are nuisance parameters and ${\theta }_{i}$ is the treatment effect for study $i$. Note that ${\theta }_{i}$ is equal to logarithm of the odds ratio for treatment compared with control. So in our cases, the negative values of ${\theta }_{i}$ represent the positive effect of the vaccination while the zero values indicate no effect.

We used the two-stage Bayesian hierarchical modeling proposed in Lunn *et al*.^28^. In the first stage, it is assumed that ${\xi }_{i}\,$s are independent from $Normal(0,{100}^{2})$ and ${\theta }_{i}\,$s are independently distributed as $Normal(0,{100}^{2})$. Also, in the second stage the underlying distributions are

3 $${{\theta }}_{{i}}|{\mu },{{\sigma }}^{2} \sim \,{Normal}({\mu },{{\sigma }}^{2}),$$

4 $${\mu }\sim {Normal}{(0,\mathrm{100}}^{{\rm{2}}}),$$

5 $${{\sigma }}^{{\rm{2}}}{ \sim }\,{Uniform}(0,100).$$

Based on the above distributions for $${\boldsymbol{\theta }}=({\theta }_{1},\ldots ,{\theta }_{N})$$, the conditional distribution of $$\mu $$ given $$p({\sigma }^{2}|\mu ,{\boldsymbol{\theta }},y)$$$$({\sigma }^{2},{\boldsymbol{\theta }},y)$$, that is $$p(\mu |{\sigma }^{2},{\boldsymbol{\theta }},y)$$, and the conditional distribution of $$\sigma $$ given $$(\mu ,{\boldsymbol{\theta }},y)$$, that is, are obtained as follows

6 $$p(\mu |{\sigma }^{2},{\boldsymbol{\theta }},y)\propto p(\mu )\prod _{i=1}^{N}p({\theta }_{i}|\mu ,{\sigma }^{2})$$

7 $$\propto \,\frac{1}{100\sqrt{2\pi }}{e}^{-\frac{1}{2\times {10}^{4}}{\mu }^{2}}\prod _{i=1}^{N}\frac{\sigma }{\sqrt{2\pi }}{e}^{-\frac{{\sigma }^{2}}{2}{({\theta }_{i}-\mu )}^{2}}$$

8 $$\,\propto \,\exp \{-\frac{1}{2}({10}^{-4}+N{\sigma }^{2})({\mu }^{2}-2\frac{{\sum }_{i=1}^{N}{\theta }_{i}}{{10}^{-4}+N{\sigma }^{2}}\mu )\}$$

This implies that the conditional distribution of $\mu$ given $({\sigma }^{2},{\boldsymbol{\theta }},y)$ is normal with mean $\frac{{\sum }_{i=1}^{N}{\theta }_{i}}{{10}^{-4}+N{\sigma }^{2}}$ and variance ${({10}^{-4}+N{\sigma }^{2})}^{-1}$. Also

9 $$p({\sigma }^{2}|\mu ,{\boldsymbol{\theta }},y)\propto p({\sigma }^{2})\prod _{i=1}^{N}p({\theta }_{i}|\mu ,{\sigma }^{2})$$

10 $$\propto \,\frac{1}{10}{I}_{(0,100)}({\sigma }^{2})\prod _{i=1}^{N}\frac{\sigma }{\sqrt{2\pi }}{e}^{-\frac{{\sigma }^{2}}{2}{({\theta }_{i}-\mu )}^{2}}$$

11 $$\,\propto \,{({\sigma }^{2})}^{\frac{N}{2}}\exp \{-\frac{{\sigma }^{2}}{2}\sum _{i=1}^{N}{({\theta }_{i}-\mu )}^{2}\}{I}_{(0,100)}({\sigma }^{2}).$$

Therefore the conditional distribution of ${\sigma }^{2}$ given ($\mu ,{\boldsymbol{\theta }},y)$ is a truncated gamma distribution on the interval $(0,100)$.

## Electronic supplementary material

Supplementary Information
