# Supplementary material for: Pneumococcal Disease and the Effectiveness of the PPV23 Vaccine in Adults: A Two-Stage Bayesian Meta-Analysis of Observational and RCT Reports
Source: Sci Rep. 2018 Jul 23;8:11051. doi: 10.1038/s41598-018-29280-2 (PMC6056566; doi:10.1038/s41598-018-29280-2)
Supplement: Supplementary file 1 — Supplementary Information [file 41598_2018_29280_MOESM1_ESM.pdf]

Pneumococcal Disease and the Effectiveness of the PPV23 Vaccine in Adults: A Two-Stage Bayesian Meta-Analysis of Observational and RCT Reports

Hamid Latifi-Navid[1], Saeid Latifi-Navid[2,3*], Behdad Mostafaiy[4], Sadegh Azimzadeh Jamalkandi[5], Ali Ahmadi[1*]

[1]*Molecular Biology Research Center, Systems Biology and Poisonings Institute, Baqiyatallah University of Medical Sciences, Tehran, Iran*
[2]*Department of Biology, Faculty of Sciences, University of Mohaghegh Ardabili, Ardabil, Iran*
[3]*Biosciences and Biotechnology Research Center (BBRC), Faculty of Advanced Technologies, University of Mohaghegh Ardabili, Namin, Iran*
[4]*Department of Statistics, Faculty of Sciences, University of Mohaghegh Ardabili, Ardabil, Iran*
[5]*Chemical Injuries Research Center, Systems Biology and Poisonings Institute, Baqiyatallah University of Medical Sciences, Tehran, Iran*

***Corresponding author: Ali Ahmadi, Ph.D.,** Applied Microbiology Research Center, Baqiyatallah University of Medical Sciences, Tehran 19395-5487, Iran, Telefax: +98218248256, E-mail: ahmadi1919@gmail.com*

***Co-corresponding author: Saeid Latifi-Navid, Ph.D. of Medical Molecular Genetics**, Department of Biology, Faculty of Sciences, University of Mohaghegh Ardabili, Ardabil, 56199-11367 Iran, Telefax: +98-45-33514701, E-mail: s_latifi@uma.ac.ir*

Supplementary Table S1. Characteristics of the RCTs of the 23-valent pneumococcal polysaccharide vaccine included in the meta-analyses

Reference	Country-year	Patient population	Number of patient	Vaccine type	Duration of follow up, years	Quality	All-cause Pneumonia	Pneumococcal pneumonia	Death from pneumonia	Invasive pneumococcal diseases
**Ortqvist et al.**	Sweden-1998	Non-immunocompromised patients aged 50 to 85 years with previous history of community acquired pneumonia	691	23 valent/placebo	5	Double - blind	63/339<br>57/352	19/339<br>16/352	2/339<br>3/352	1/339<br>5/352
**French et al.**	Uganda-2000	HIV-1 infected Ugandans <55 years (about 14% with previous history of pneumonia)	1323	23 valent/placebo	2.7	Double - blind	40/667<br>21/656	20/667<br>14/656	NA	15/667<br>10/656
**Watera et al.**	Uganda-2004	HIV-infected Ugandan adults	473	23 valent/placebo	6	Double - blind	51/260<br>32/213	35/260<br>27/213	NA	30/260<br>22/213
**Alfageme et al.**	Spain-2006	immunocompetent patients with chronic obstructive pulmonary disease	596	23 valent	2.7	Open (No intervention used in control group)	25/298<br>33/298	0/298<br>5/298	6/298<br>6/298	NA

**Maruyama et al.**	Japan-2010	using this test in nursing home residents in a rural area of Japan showed an incidence of pneumococcal pneumonia about 20 times higher than in the elderly community dwelling population	1006	23 valent/placebo	2.2	Double-blind	63/502<br>104/504	14/502<br>37/504	13/502<br>26/504	0/502<br>3/504
**Russell et al.**	America-2015	healthy military trainees aged 18–20	152,723	23 valent/placebo	6.7	Double-blind	180/76245<br>188/76478	NA	NA	NA

NA: not available

Supplementary Table S2. Characteristics of the cohort studies of the 23-valent pneumococcal polysaccharide vaccine included in the meta-analyses.

Reference	Country-year	Case	Control	Number of Case (Male number)	Number of Control (Male number)	Age, years	Duration of follow up, years	All-cause pneumonia Case/Control	Pneumococcal Pneumonia Case/Control	Pneumonia death Case/Control	Invasive pneumococcal diseases Case/Control	Quality
**Christenson et al.**	Stockholm County-2001	All individuals in Stockholm County aged 65 years or older (259 627) were invited to take part in a vaccination campaign		841 (NA)	159385 (61994)	≥65	3	5/841 2468/159385	0/841 80/159385	NA	0/841 33/159385	prospective study
**Lo´pez-Palomo et al.**	Spain-2004	HIV-1-infected patients had received vaccine	HIV-1-infected patients had not been vaccinated	159 (1.3:1)	141 (1.2:1)	37±7	2.9	14/159 26/141	3/159 9/141	NA	1/159 6/141	Cohort Study
**Vila-Co´rcoles et al. 1**	Spain (Tarragona, Catalonia)-2005	all individuals assigned to one of eight primary care centres in Tarragona, Spain		4986 (2176)	6255 (2716)	≥65	3	NA	NA	6/4986 12/6255	NA	prospective cohort study
**Mykietiuk et al.**	Spain (Barcelona Catalonia) 2006	Vaccinated patients with community-acquired pneumococcal pneumonia	unvaccinated patients with community-acquired pneumococcal pneumonia	61 (44)	438 (294)	Unvaccinated:65.1±16.7 Vaccinated 74.2±13.0	13 February 1995 through 31 December 2004	NA	NA	NA	10/61 153/438	Cohort

**Vila-Co´ rcoles et al. 2**	Spain (Tarragon a) -2006	all community-dwelling subjects assigned to 1 of 8 primary health care centers (PHCCs) in Tarragona, Catalonia, Spain	4986 (2176)	6255 (2716)	≥65	January 2002 through April 2005	NA	26/4986 26/6255	26/4986 34/6255	8/4986 14/6255	prospect ive cohort study
**Johnstone et al.**	**Canada-2010**	adults with CAP admitted to the hospital in Edmonton, Alberta, Canada	956	1994	≥65	2000 - 31 March 2006	NA	NA	528/956 876/1994	NA	cohort
**Siemieniuk et al.**	**Southern Alberta, Canada 2011**	in a regional HIV population in Southern Alberta, Canada	472	429	NA	January 1st, 2000 and January 1st, 2010	NA	12/472 2/429	NA	12/472 9/429	cohort
**Ochoa-Gondar et al.**	**Tarragona, Spain 2014**	individuals in Tarragona, Spain	8981 (4063)	12044 (5454)	≥60	1 December 2008 until 30 November 2011	207/8981 359/12044	NA	27/8981 48/12044	4/8981 12/12044	cohort
**Gutiérrez Rodríguez et al.**	**Madrid, Spain 2014**	IPDs registered in the Surveillance System of the Region of Madrid	368 (220)	496 (260)	≥60	2008–2011	NA	223/368 314/496	NA	51/368 74/496	cohort
**Tsai et al.**	**Taiwan-2015**		229181 (119336)	229181 (119545)	≥75	NA	NA	NA	153/229181 2168/229181	11/229181 46/229181	

NA: not available

Supplementary Table S3. Characteristics of the case-control studies of the 23-valent pneumococcal polysaccharide vaccine included in the meta-analyses

Reference	Country- year	Case	Control	Number of Case (Male number)	Number of Control (Male number)	Age, years	All-cause pneumonia Case/Control	Pneumococcal Pneumonia Case/Control	Death from pneumonia Case/Control	Invasive pneumococcal diseases Case/Control	Quality
**Breiman et al.**	USA-2000	Subjects with HIV infection Whom Streptococcus pneumonia was isolated from a normally sterile site.	HIV infected patients or clinical stage of acquired immunodeficiency syndrome	176 (147)	327 (269)	18-55	NA	NA	NA	41/176 112/327	Case-control
**Benin et al.**	USA (Navajo adults)-2003	A case patient was defined as a Navajo adult who was registered and who had invasive pneumococcal diseases in 1996 or 1997	were chosen from randomly-selected medical records of adult Navajo registered	108 (60)	330 (182)	41-71	NA	NA	NA	67/108 211/330	Case-Control

**Amelia SM Veras et al.**	Brazil (São Paulo) -2007	A case was defined as any HIV-infected individual over 18 years of age with invasive pneumococcal diseases	Controls were defined as HIV-infected individuals over 18 years of age, without a history of documented or suspected invasive pneumococcal diseases	79 (49)	242 (154)	≤38 ≥ 39	NA	NA	NA	14/79 85/242	Case – control
**Imaz et al.**	Spain- 2009	HIV-infected adult patients hospitalized with invasive pneumococcal diseases that receive vaccine	HIV-infected adult patients hospitalized with invasive pneumococcal diseases that don't receive vaccine	23 (20)	139 (106)	38.5	NA	NA	0/23 13/139	21/23 113/139	Case – control
**Vila-Corcoles et al. 3**	Tarragona,Spain-2010	A case was defined as a patient aged 60 years or older, living in the study area, who had a laboratory-confirmed episode of IPDs during the study	control subjects who were matched by primary care centre, age, sex and risk stratum	88 (54)	176 (108)	≥60	NA	NA	NA	34/88 104/176	Case – control

	period

NA: not available

Supplementary Table S4. Meta-analysis of the effectiveness of PPV-23 on invasive pneumococcal disease, including all the cohort/case-control studies: "Leave-One-Out" sensitivity analysis.

	Random-effects model			Test of homogeneity		Publication bias *P*	
**Studies**	**RR (95% CI)**		***P***	I^2	τ^2	**Egger's regression Intercept *P***	**Begg' *P***
**Breiman et al.**	0.702	(0.543-0.906)	0.007	75.558	0.106	0.0365	0.731
**Benin et al**	0.655	(0.491-0.875)	0.004	76.402	0.147	0.0442	0.945
**Amelia SM Veras et al**	0.727	(0.572-0.924)	0.009	74.531	0.092	0.0493	0.731
**Imaz et al**	0.652	(0.507-0.838)	0.001	66.785	0.096	0.0925	0.537
**Vila-Corcoles et al 1**	0.707	(0.548-0910)	0.007	74.692	0.103	0.0320	0.537
**Christenson et al**	0.695	(0.549-0.880)	0.002	76.830	0.099	0.0082	0.303
**Vila-Corcoles et al 2**	0.700	(0.549-0.892)	0.004	77.037	0.102	0.0327	0.631
**Siemieniuk et al**	0.681	(0.534-0.868)	0.002	76.900	0.101	0.0157	0.537
**Tsai et al**	0.774	(0.631-0.951)	0.015	66.254	0.061	0.0550	0.945
**Lo´pez-Palomo et al**	0.716	(0.568-0.903)	0.005	75.743	0.094	0.0518	0.731
**Mykietiuk et al**	0.729	(0.574-0.924)	0.009	74.663	0.092	0.0519	0.631
**Ochoa-Gondar et al**	0.713	(0.562-0.904)	0.005	76.455	0.098	0.0431	0.731
**Gutiérrez Rodríguez et al**	0.672	(0.517-0.873)	0.003	77.110	0.114	0.0337	0.837

Supplementary Table S5. Quality ratings for the 6 RCTs included on the basis of Jadad assessment scale

Study	Randomization		Blinding		An account of all patients	result
	randomization is mentioned	randomization is appropriate or inappropriate	blinding is mentioned	the method of blinding is appropriate or inappropriate	The fate of all patients in the trial is known. If there are no data the reason is stated.	
**Ortqvist et al.**	+1 (Patients who had been treated in hospital for community-acquired pneumonia (CAP) were randomly assigned in equal proportions)	+1 (Randomization was done by the vaccine manufacturer (MSD) and the code was not disclosed to the investigators until follow-up had ended. Random numbers (1 to 1050) were allocated to vials containing vaccine or placebo, and balanced within each center. Vials were then taken in chronological order for vaccination of consecutive patients.)	+1	+1 (Random numbers (1 to 1050) were allocated to vials containing vaccine or placebo, and balanced within each center. Vials were then taken in chronological order for vaccination of consecutive patients.)	+1	+5
**French et al.**	+1(Randomization took place in groups of 20 and was done independently for the two study clinics.)	+1 (Randomization was successful for the remaining 1323 participants with baseline demographic characteristics of the vaccine and placebo recipients well matched (table 1).	+1	+1 (Vaccine and placebo were presented in identical single dose syringe and needle combinations, labelled with sequential study numbers only.)	+1	+5

**Watera et al.**	0	0	+1	+1 (Both the participants and the trial staff remained blind to the vaccine status.)	+1	+3
**Alfageme et al.**	+1 (They were then randomly assigned to the intervention group and received 23-valent pneumococcal capsular polysaccharide vaccine (Pneumo 23; Aventis Pasteur MSD), together with a clinical follow up examination.	+1 (A randomization code was developed using a computer random number generator in block lengths of 20 (10 in each group)	0 (A considerable limitation of this study is the lack of a blind placebo comparison group. Nevertheless, it is highly unlikely that this limitation significantly influenced the results because the vaccination status of the patient was kept in a specific encrypted database and was not stated in the patients' clinical records.)	0	+1	+3
**Maruyama et al.**	+1 (We randomly assigned participants to receive either 0.5 ml (25 µg in each) 23-valent pneumococcal polysaccharide vaccine (Pneumovax, Merck, NJ) or 0.5 ml placebo (sodium chloride) intramuscularly)	+1 (A statistician who was not on the study team carried out the randomization using a random number table, and numbered the containers.)	+1	+1 (Vaccine and placebo were presented in identical single dose syringe and needle combinations, labelled with sequential study numbers only.)	+1	+5
**Russell et al.**	+1 (Study participants completed a study questionnaire and were administered a prepackaged, blinded, and randomized intramuscular deltoid injection	+1 (Randomization was conducted by a third party in a simple 1:1 ratio, and tubes were labeled with a unique identifier)- The randomization	+1	+1 (Study participants completed a study questionnaire and were administered a prepackaged, blinded, and randomized intramuscular deltoid injection containing either the PPV23	+1	+5

	containing either the PPV23 (Wyeth Pharmaceuticals or Merck & Co., Inc.) or saline.)	process generated vaccine and placebo treatment groups that were relatively balanced by demographic characteristics	(Wyeth Pharmaceuticals or Merck & Co., Inc.) or saline.)

Supplementary Table S6. Quality ratings for the 10 cohort studies included on the basis of Newcastle-Ottawa quality assessment scale.

	Selection (score)				Comparability (score)	Outcome (score)			Total Score
	Representative of exposed cohort	Selections of non-exposed cohort	Assessment of exposure	Absence of outcome at start of study	Control for age, sex, etc.	Assessment of outcome	Follow-up period (≥ 5 years)	Adequacy of follow-up	
Christenson et al. 2001	1	1	1	1	2	1	0	1	8 (high)
Lo ´pez-Palomo et al.2004	1	1	1	1	2	1	0	1	8 (high)
VilaCo´rcoles et al. 1-2005	1	1	1	1	2	1	0	1	8 (high)
Mykietiuk et al. 2006	1	1	1	1	2	0	1	1	8 (high)
Vila-Co´rcoles et al. 2- 2006	1	1	1	1	2	1	0	0	7 (high)
Johnstone et al. 2010	1	1	1	1	2	1	1	0	8 (high)
Siemieniuk et al. 2011	1	1	1	1	2	0	1	0	7 (high)
Ochoa-Gondar et al. 2014	1	1	1	1	2	1	0	0	7 (high)
Gutiérrez Rodríguez et al. 2014	1	1	0	1	2	1	0	1	7 (high)
Tsai et al. 2015	1	1	0	1	2	1	0	0	6 (low)
								Mean	7.4

Supplementary Table S7. Quality ratings for the 5 case-control studies included on the basis of Newcastle-Ottawa quality assessment scale.

	Selection (score)				Comparability (score)	Exposure (score)			Total Score
	Case definition	Representative of cases	Selections of controls	Definition of controls	Control for age, sex, etc.	Ascertainment of exposure	Same method of ascertainment for participants	Nonresponse rate	
Breiman et al. 2000	1	1	1	1	2	1	1	0	8
Benin et al. 2003	1	1	1	1	2	1	1	1	9
Amelia SM Veras et al. 2007	1	1	1	1	2	1	1	1	9
Imaz et al. 2009	1	1	1	1	2	1	1	0	8
VilaCorcoles et al. 3-2010	1	1	1	1	2	1	1	1	9
								Mean	8.6

Supplementary Table S8. Detailed information about *methods* which were used for the diagnosis of pneumonia

	RCTs
**Örtqvist et al, 1998**	1:Clinical plus X-ray<br>2:Pneumonia plus culture or serology - Primary endpoints were pneumonia, defined as acute clinical symptoms or signs compatible with a lower respiratory-tract infection and a new infiltrate on chest radiography;
**French et al, 2000**	1:All definite and probable invasive pneumococcal disease events isolation of *S pneumoniae* from a normally sterile site—ie, blood, cerebrospinal fluid, pleural fluid, or pus (but not mucous membranes)
**Watera et al, 2004**	-
**Alfageme et al 2006**	Patients admitted to hospital were given diagnostic tests as considered necessary by their responsible doctor, according to the seriousness of the case and in order to arrive at a diagnosis (for example, blood culture, thoracocentesis). All patients with an initial diagnosis of pneumonia were subject to a new regime which included clinical revision and radiography 2–4 weeks after the first visit. All the radiographs were examined by two doctors participating in the study.
**Maruyama et al. 2010**	Pneumonia was diagnosed by the medical staff of the respiratory unit at the affiliated hospital on the basis of the presence of clinical symptoms and a new infiltrate on chest radiography Pneumococcal pneumonia was diagnosed from a positive result in blood culture, pleural fluid, or sputum (107 colony forming units per millilitre in a purulent sample) or a positive pneumococcal antigen test result in urine.
**Russell et al. 2015**	Radiographically confirmed during the recruit training period
	**Cohort stud**ies
**Christenson et al 2001**	All individuals (65 years) admitted to hospital in Stockholm County with influenza and pneumonia related diagnoses were identified between Dec 1, 1998, and May 31, 1999.
**Lo ´pez-Palomo 2004**	Patients were considered as having a pneumonia if they met the following three criteria: (1) presence of fever (temperature >388C), dyspnea, cough, and/or<br>expectoration; (2) a new pulmonary infiltrate in a chest radiograph, for which noninfectious causes were excluded; and

	either (3a) a diagnosis based on at least one of the following samples: blood, pleural fluid, sputum with culture, bronchial aspirate (106 cfu/ml), bronchoalveolar lavage fluid (104 cfu/ml), protected brush specimen (103 cfu/ml), transbronchial biopsy or pulmonary needle aspirate; or (3b) the presence of the first two criteria and a cure with antibacterial treatment (other than cotrimoxazole)
**Vila-Corcoles et al., 2005**	All pneumonias were validated by checking clinical records. In all the cases the medical records were reviewed to validate the diagnosis using the same criteria as for hospitalised pneumonia. In all cases, the presence of an infiltrate on chest radiographs was necessary to validate the diagnosis.
**Mykietiuk et al. 2006**	Pneumococcal pneumonia was diagnosed in patients with signs and symptoms of an acute-onset lower respiratory tract infection, a new infiltrate on chest radiograph, and one or more cultures positive for S. pneumoniae obtained from blood, normally sterile fluids, or sputum and/or a positive test for detection of urinary antigen.
**Vila-Corcoles et al. 2006**	Discharge diagnoses, with information from laboratory data on S. pneumoniae sterile site isolates
**Johnstone et al. 2010**	presence of 2 signs or symptoms of CAP (cough [productive or nonproductive], pleuritic chest pain, shortness of breath, temperature >38°C, and crackles or bronchial breathing on auscultation), plus radiographic evidence as determined by the treating physician.
**Siemieniuk et al. 2011**	Pneumococcal pneumonia was defined as isolation from a sputum sample or via bronchoalveolar lavage (BAL); most BAL isolates were also serotyped. Sputum and BAL samples were obtained because of strong clinical suspicion for lower respiratory tract infection, generally associated with radiologic evidence and severe illness.
**Ochoa-Gondar et al. 2014**	All CAP cases were radiographically confirmed and validated by checking clinical records Hospital databases for discharge diagnoses and medical records of an acute respiratory illness with a new infiltrate on chest radiography.
**Gutierrez-Rodriguez et al., 2014**	Laboratory data on S. pneumoniae sterile site isolates, PCR detection or antigen detection
**Tsai et al., 2015**	Database records of discharge diagnoses

Case-control studies	
**Breiman et al. 2000**	A case of invasive pneumococcal disease was defined as the isolation of S pneumoniae from a normally sterile site, including blood, cerebrospinal fluid, pleural fluid, open or transthoracic lung biopsy material and joint aspirate material. Cases were identified from records in the microbiology laboratories at participating hospitals
**Benin et al. 2003**	Invasive pneumococcal disease was defined as isolation of *Streptococcus pneumoniae* from a normally sterile body fluid, such as blood or cerebrospinal fluid. Case patients were identified through an existing system of active, laboratory-based surveillance conducted by the Johns Hopkins Center for American Indian Health (Baltimore, MD)
**Amelia SM Veras et al. 2007**	documented S. pneumoniae infection of a normally sterile site, such as blood or cerebrospinal fluid
**Imaz et al. 2009**	Invasive pneumococcal pneumonia was diagnosed when a patient had consistent clinical findings plus a new pulmonary infiltrate on chest radiography and isolation of S. pneumoniae in blood and/or pleural fluid cultures.
**Vila-Corcoles et al., 2010**	Database records of discharge diagnoses, and additional information from laboratory data on S. pneumoniae sterile site isolates
